# Efficacy and safety of ultrasound-guided percutaneous microwave ablation for the treatment of hepatic alveolar echinococcosis: A preliminary study

**DOI:** 10.1097/MD.0000000000007137

**Published:** 2017-07-07

**Authors:** Yangdan Cairang, Lingqiang Zhang, Bin Ren, Li Ren, Lizhao Hou, Haijiu Wang, Ying Zhou, Qingxi Zhang, Jun Shao, Haining Fan

**Affiliations:** a Department of Hepatopancreatobiliary Surgery, the Affiliated Hospital of Qinghai University; b Qinghai Province Key Laboratory of Hydatid Disease Research; c Medical College of Qinghai University, Xining, China.

**Keywords:** echinococcus granulosus, hepatic alveolar echinococcosis, microwave ablation

## Abstract

The present study aims to assess the efficacy and safety of ultrasound-guided percutaneous microwave ablation (MWA) for hepatic alveolar echinococcosis (HAE) preliminarily.

Seventeen patients diagnosed to HAE and treated with MWA (80 watts, 4 min) were retrospectively analyzed. The upper abdominal computed tomography (CT) was performed at 1, 6, 12 months after the MWA treatment. The complications were evaluated to assess the safety.

The diameters of the lesions in the HAE patients ranged from 1.9 to 4.7 cm. The patients included 10 males and 7 females, aged 26 to 70 (45.82 ± 13.36) years, 5 patients infecting with chronic hepatitis viral B and 8 patients with positive hydatid antibody (IgG). The lesions observed in the postoperative CT (1, 6, 12 months) were calcified compared with those observed in the preoperative CT and without relapse. No serious treatment-related complications occurred after treatment.

MWA is a novel and effective therapeutic method for HAE with a single lesion (diameter≤=5 cm). Further studies based on prospective random control trials to confirm our findings are necessary.

## 1 Introduction

Hepatic alveolar echinococcosis (HAE) is a serious zoonotic parasitic disease. As intermediate hosts, human beings are infected directly through close contacting with definitive hosts or indirectly through food or water contaminated with parasite eggs.^[1]^ In recent years, the incidence of HAE increased in Chinese northern-western regions, especially in the Qinghai province, where the incidence has increased to 0.63%. HAE is similar to a malignancy, showing infiltrative growth and distant metastasis that resulted in mechanical compression and an inflammatory reaction.^[2]^ Most of the patients with HAE suffer from abdominal pain, jaundice, and even liver failure. Surgical resection or liver transplantation can produce good outcomes for HAE patients with large lesions. However, with HAE lesions≤5 cm, the treatment effect of drugs (such as albendazole) is poor. Therefore, the further exploration of novel treatment methods for early-stage HAE is necessary.

Microwave ablation (MWA), also known as microwave coagulation or percutaneous microwave coagulation therapy, has been widely used to treat liver cancer.^[3–7]^ However, the treatment effects of MWA for HAE have not been reported at present. Therefore, this study aimed to assess the therapeutic effects of MWA for HAE.

## 2 Subjects and methods

### 2.1 Subjects

A total of 17 HAE patients treated with MWA in the Affiliated Hospital of Qinghai University were retrospectively analyzed. The clinical information is shown in Table 1. The patients included 5 individuals with chronic hepatitis viral B and 8 individuals with positive hydatid antibody (LgG). The inclusion criteria were shown as follows: considering to diagnose to HAE based on pastoral life history, right upper abdominal pain, and imaging detection results (such as computed tomography (CT) scan or magnetic resonance imaging); the lesions diameters were less than 5 cm. The exclusion criteria were: the lesion >5 cm or multiple lesions in the liver; imaging detection indicated metastatic lesions in other tissues and organs; the patients could not tolerate the operation since severe cardiopulmonary dysfunction; severe coagulation dysfunction. The present study was approved by the Ethics Committee of the Affiliated Hospital of Qinghai University Hospital. All the participants signed written consent and the records of study participants were anonymized.

**Table 1:**
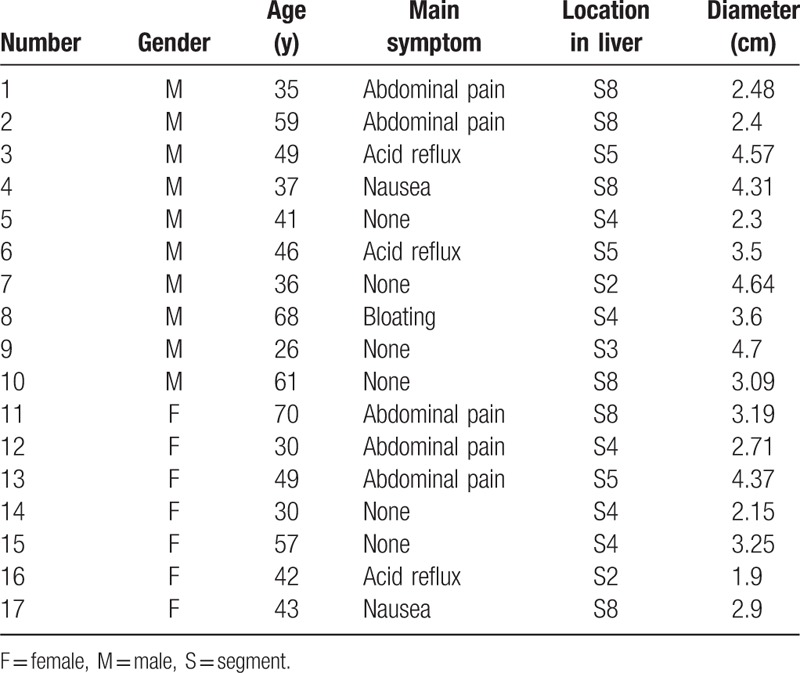
The clinical characteristics of the 17 HAE patients.

### 2.2 The treatment process

The microwave ablation apparatus (Nanjing Kang You Medical Technology Co, Ltd, Nanjing, China) was used to treat HAE (output frequency 2450 MHZ; output power 0–120 watts). A portable color Doppler ultrasound apparatus (M7 Series; the ultrasound probe model, 3C5s) with a puncture guide bracket was then used to locate the HAE lesions. Pethidine (50 mg), diazepam (10 mg), or hydrochloride promethazine (25 mg) were intramuscularly injected preoperatively. After disinfection, anesthesia, and the placement of sterile towels, the outer layer of the HAE lesion was located by ultrasound and was then punctured by a microwave antenna. After reaching the lesion position, the microwave apparatus was set to 80 watts, 4 minutes. During the ablation process, we can observe a strong echo in the B-ultrasound indicated that the lesion has been carbonized. After finishing the operation, the wound was disinfected and covered with the dressing.

### 2.3 Postoperative observation

The patients were required to regularly review the upper abdominal CT. The presence of a reaction zone around MWA zone at 1 year postoperative was considered to represent a recurrence of HAE. The complication after surgery was also closely observed.

## 3 Results

### 3.1 The clinical characteristics of the HAE patients treated with MWA

The clinical characteristics for the 17 HAE patients treated with MWA are shown in Table 1. The objectives included 10 (58.82%) male and 7 (41.18%) female patients. The age range was from 26 to 70 (45.82 ± 13.36). Of these, 5 cases (29.41%) presented with abdominal pain as the main symptom, 3 cases (17.65%) with nausea, 3 (17.65%) cases with acid reflux, and the remainder without obvious symptoms. At presentation, the diameter of the lesions ranged from 19 to 4.7 cm (3.0 ± 0.94). Most of the HAE lesions were detected in segment 8 (35.29%) and 4 (29.41%) of the liver, while the other lesions were found in segments 5, 3, and 2 of the livers.

### 3.2 The treatment effects of MWA for HAE

**Figure 1:**
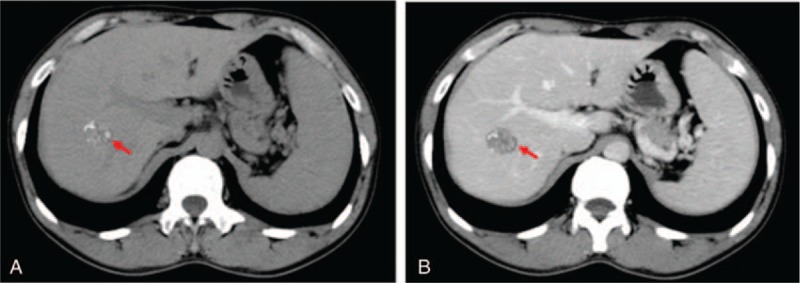
The preoperative upper abdominal CT for the HAE patients. A, Unenhanced CT. B, Enhanced CT. The red arrow indicates the HAE lesion. HAE = hepatic alveolar echinococcosis, CT = computed tomography.

**Figure 2:**
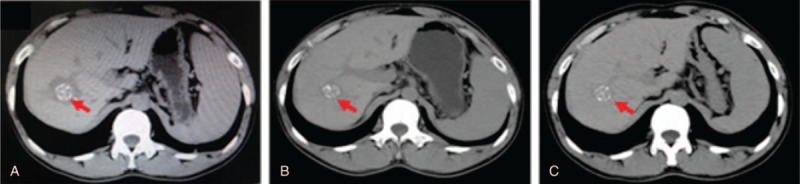
The computed tomography (CT) scan of the upper abdomen for the HAE patients at 1, 6, 12 months after the microwave ablation treatment. A, CT scan 1 month after surgery. B, CT scan 6 months after surgery. C, CT scan 12 months after surgery. The red arrow indicates the HAE lesion. HAE = hepatic alveolar echinococcosis.

The HAE lesions were located mainly in the right lobe of the liver. The preoperative upper abdominal CT (unenhanced and enhanced) for the HAE patients is shown in Figure 1. After the treatment with MWA at 1, 6, and 12 months (shown in Fig. 2), the upper abdominal CT detection for the HAE patients indicated that there were no incomplete ablation, local recurrence, or death. The percentage of the complete ablation was 100%.

### 3.3 The safety assessment of MWA treatment for HAE

After MWA treatment, there were 1 patient with a high fever and 1 patient with abdominal pain. All the symptoms disappeared after 1 week postoperatively for all the 17 patients. Other complications, such as bile leakage, hemorrhage, and infection, were not observed (Table 2). The liver function detection showed a slight increase after surgery, with a recovery after 2 weeks (Table 3).

**Table 2:**
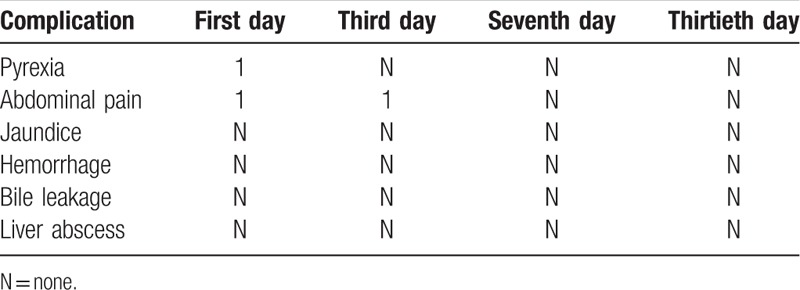
Complications after MWA treatment.

**Table 3:**
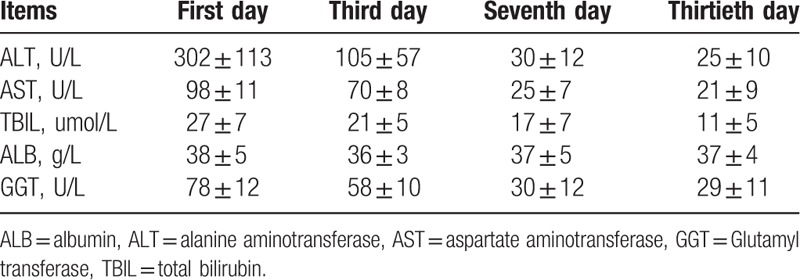
The liver function detection after MWA treatment.

## 4 Discussion

Hepatic hydatid disease was mainly divided into 2 types: cystic echinococcosis and alveolar echinococcosis. These are caused by echinococcosis granulosus and echinococcosis multilocularis, respectively. Given the similar nature as malignant tumors and the absence of special symptoms, the majority of HAE patients are diagnosed in a late stage, after the most favorable time for surgery has passed. The 10-year mortality rates for HAE were 75% to 94%, which seriously affect the safety of the lives and property of the local people.

At present, there are several treatment methods for HAE, such as chemotherapy (albendzole), laparoscopic therapy, puncture percutaneous biliary drainage, radiofrequency ablation, liver transplantation, and so on.^[8–11]^ Surgical resection or liver transplantation remain the preferred choices for HAE patients. According to the literature report, the recurrence rate of HAE after open-abdominal surgery was 16.5% to 33%. The recurrence rate of surgical drainage and anhydrous injection therapy was 18.5% to 36%. However, the surgical treatment of early-stage HAE remains controversial.

The application of MWA was first reported by Seki et al^[12]^ and has been applied widely to treat primary hepatocellular carcinoma. Although the friction of intracellular and extracellular charged ions is due to the microwave frequency and collisions with other molecules to produce heat, the local temperature exceeds 65 °C over a short period of time, producing coagulation necrosis. Additionally, MWA could improve immune function by stimulating Th1 cell production.^[13]^ However, it is unclear if MWA is effective and safe for the treatment of HAE. The present study indicated that the HAE lesions gradually calcify after MWA treatment without serious complications or recurrence after surgery. Therefore, MWA may be a novel therapeutic method for the treatment of early-stage HAE with lesion diameters <5 cm.

Some limitations in the present study should be taken into account. Our study was performed retrospectively, such that the small sample size may affect our conclusions. Therefore, larger, multicenter, prospective studies are required to validate our findings.

In conclusion, MWA is a novel treatment method that is effective and safe for early-stage HAE. Despite this, our findings should be confirmed by larger prospective studies completed in multiple centers.
